# Fenpropathrin induces degeneration of dopaminergic neurons via disruption of the mitochondrial quality control system

**DOI:** 10.1038/s41420-020-00313-y

**Published:** 2020-08-25

**Authors:** Zhigang Jiao, Yixuan Wu, Shaogang Qu

**Affiliations:** 1 Department of Neurology, Nanfang Hospital, Southern Medical University, Guangzhou, 510515 Guangdong China; 2 Central Laboratory and Department of Neurology, Shunde Hospital, Southern Medical University (The First People’s Hospital of Shunde Foshan), Foshan, 528300 Guangdong China; 3 Guangdong-Hong Kong-Macao Greater Bay Area Center for Brain Science and Brain-Inspired Intelligence, Guangzhou, 510515 Guangdong China; 4 School of Basic Medical Sciences, Southern Medical University, Guangzhou, 510515 Guangdong China

**Keywords:** Diseases, Parkinson's disease, Diseases, Parkinson's disease

## Abstract

The synthetic pyrethroid derivative, fenpropathrin, is a widely used insecticide. However, a variety of toxic effects in mammals have been reported. In particular, fenpropathrin induces degeneration of dopaminergic neurons and parkinsonism. However, the mechanism of fenpropathrin-induced parkinsonism has remained unknown. In the present study, we investigated the toxic effects and underlying mechanisms of fenpropathrin on perturbing the dopaminergic system both in vivo and in vitro. We found that fenpropathrin induced cellular death of dopaminergic neurons in vivo. Furthermore, fenpropathrin increased the generation of reactive oxygen species, disrupted both mitochondrial function and dynamic networks, impaired synaptic communication, and promoted mitophagy in vitro. In mice, fenpropathrin was administered into the striatum via stereotaxic (ST) injections. ST-injected mice exhibited poor locomotor function at 24 weeks after the first ST injection and the number of tyrosine hydroxylase (TH)-positive cells and level of TH protein in the substantia nigra pars compacta were significantly decreased, as compared to these parameters in vehicle-treated mice. Taken together, our results demonstrate that exposure to fenpropathrin induces a loss of dopaminergic neurons and partially mimics the pathologic features of Parkinson’s disease. These findings suggest that fenpropathrin may induce neuronal degeneration via dysregulation of mitochondrial function and the mitochondrial quality control system.

## Introduction

Parkinson’s disease (PD) is a neurodegenerative disease that is often manifested by tremors, rigidity, motor dysfunction, and cognitive impairment. The pathological features of PD include progressive loss of dopaminergic (DA) neurons and generation of Lewy bodies in the substantia nigra pars compacta (SNpc). Although the etiology of PD remains to be fully elucidated, environmental toxicants, such as pesticides, are considered to be a risk factor for PD^1^. Pyrethroid (Pyr) insecticides are used to control insect pests in both agriculture and veterinary medicine worldwide^2,3^. With the increasing use of these compounds, the risk of human exposure to Pyr residues in vegetables, fruits, well water, and carpets soaked to eliminate insect pests in homes has risen and potential side effects of Pyr exposure have received increased attention. Many studies have indicated that Pyr has neurotoxic effects and leads to the degeneration of neurons via disturbing the balance between oxidants and antioxidants in mouse brains, as well as by affecting the expression of dopamine transporter and/or vesicular monoamine transporter 2 in DA neurons^4–6^. Using a previous case study as a correlational example in humans, a man who had consumed fenpropathrin (Fen) derived from Fen-poisoned fish for half a year was diagnosed with parkinsonism in China^5^.

Mitochondrial function and dynamic networks are essential for maintaining the survival and development of neurons^7^. Previous studies have indicated that dysregulation of mitochondrial function and mismatches in dynamic networks are involved in the occurrence and progression of PD^8,9^, which include increased reactive oxygen species (ROS), impaired mitochondrial electron transport chains, loss of MMP, abnormal mitochondrial morphology, and abnormal mitochondrial dynamics^8,10,11^. For example, mutations of the genes encoded by PTEN-induced kinase 1 (PINK1), Parkin, and DJ-1 all cause autosomal recessive PD and have well-confirmed roles in mitochondrial homeostasis and mitophagy; specifically, PINK1 and Parkin are important activators of mitophagy, whereas DJ-1 is a chaperone and a redox sensor^12,13^. Moreover, sporadic PD can be caused by environmental toxicants such as 1-methyl-4-phenyl-1,2,3,6-tetrahydropyridine (MPTP), 6-Hydroxydopamine hydrobromide (6-OHDA), rotenone, and Fen^5,14–16^, each of which impair mitochondrial function or perturb mitochondrial dynamics^17–20^. Dynamin-related protein 1 (Drp1) and the dynamin-related GTPases, mitofusin 1 (Mfn-1) and 2 (Mfn-2), contribute to maintaining equilibrium of mitochondrial dynamics^21^. Many human diseases are caused by mutations of these GTPases. For example, mutations in Drp1 induce neurodevelopmental disorders, dynamin-2 and Mfn-2 are abnormally expressed in Charcot-Marie-Tooth neuropathy, and mutations in Opa1 result in dominant optic atrophy^22^. Moreover, several studies have indicated that the expression of Drp1 is increased in MPTP-treated mice^18,19^, and inhibition of Drp1 attenuates neurotoxicity in MPTP-treated mice^23^. Although the comprehensive roles of mitochondrial fission and fusion remain to be fully elucidated, a balance of fusion and fission is known to be crucial to mitochondrial morphology, cellular viability, and synaptic function, all of which are dysregulated in PD.

The elimination of dysfunctional and/or senescent mitochondria is accomplished via mitophagy^24^, which is activated by a number of stimuli, including hypoxia, energy stress, and increased oxidative phosphorylation. Disrupted mitochondrial function can lead to excessive mitochondrial elimination and/or progressive accumulation of defective organelles, resulting in cellular and tissue damage^25^. For instance, Chung-Han Hsieh et al.^26^ revealed that mutations in leucine-rich repeat kinase 2 or PINK1/Parkin inhibit Miro-induced removal of depolarized mitochondria and disrupt mitophagy in an in vitro fibroblast model of PD. Heterozygous mutations of glucosylceramidase beta are the most common genetic risk factor for PD, as these mutations disrupt mitochondrial function and mitophagy^27^. Together, these studies indicate that genetic- or environmental-induced dysregulation of mitophagy contributes to the development of PD.

Increasing evidence has shown that environmental factors, such as pesticides, disrupt mitochondrial function and/or mitophagy and ultimately induce neurodegeneration^28^. Pyr has been shown to be neurotoxic and to induce neuronal injury in mammals^29^; however, the mechanism of Pyr-induced impairment in neurons has remained unclear. Thus, in our current study, we investigated the underlying mechanisms of Fen-induced neuronal degeneration. We used Fen to treat primary neurons in vitro and mice in vivo. We found that neuronal loss was significantly increased following Fen treatment, both in vivo and in vitro. Interestingly, we found that Fen induced an alteration in mitochondrial size by decreasing the expression of Drp1, which led to oxidative stress—as indicated by an increase in ROS levels and production of 4-hydroxynonenal (4-HNE)—that ultimately disrupted mitochondrial function. We further found that mitophagy was enhanced due to eliminating dysfunctional mitochondria in primary neurons in vitro. We conclude that Fen impaired mitochondrial function, decreased mitochondrial mass and insufficient ATP production by inhibiting mitochondrial regeneration, and enhanced mitophagy, all of which ultimately led to disrupted synaptic transmission and neuronal degeneration.

## Results

### Fen reduces locomotor activity and induces degeneration of DA neurons in mice

**Fig. 1:**
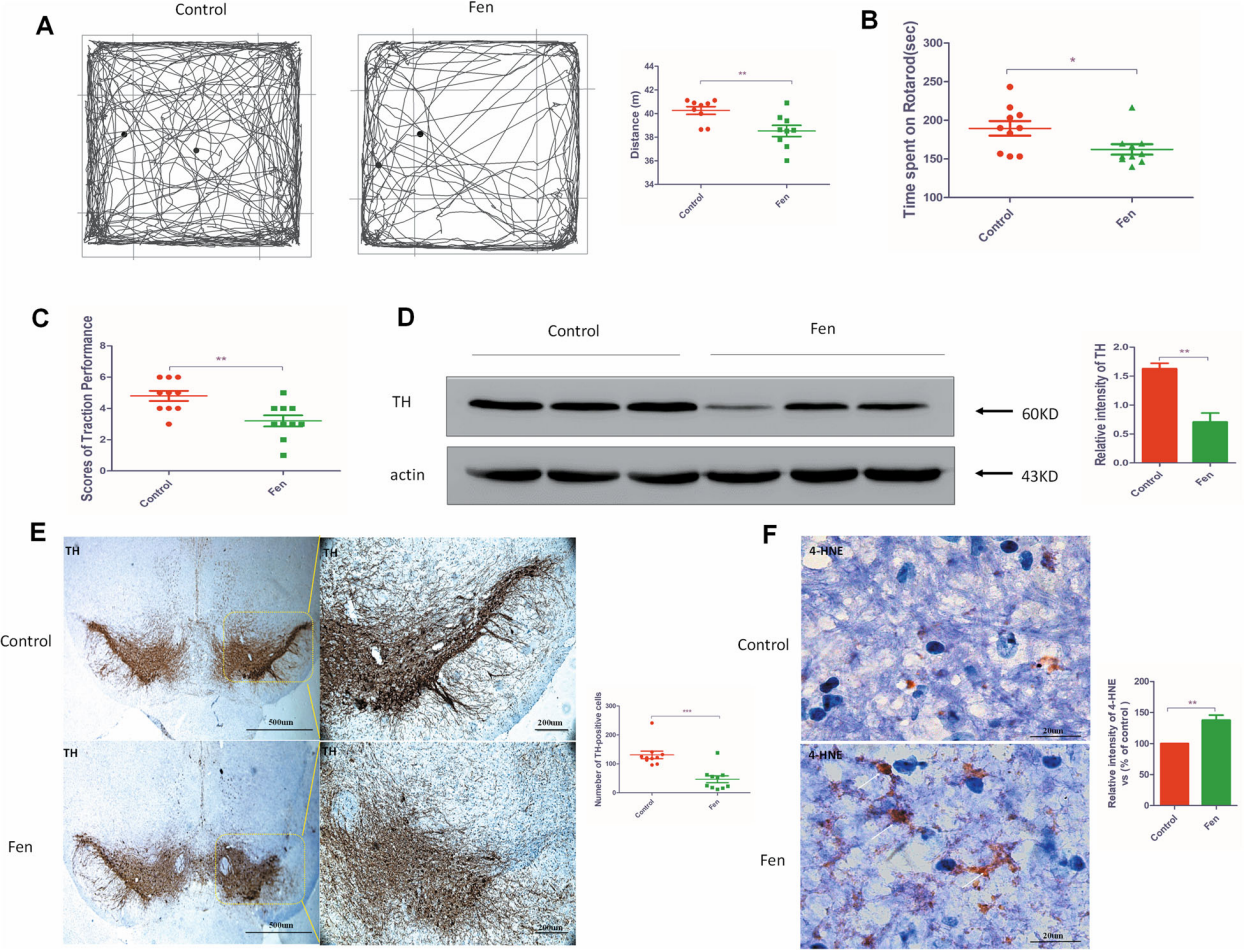
Fen induces motor deficits and degeneration of DA neurons in mice. **a** Open field tests, **b** rotarod tests, and **c** traction tests were performed at 24 weeks after the first Fen/vehicle treatment via ST injections into the striatum (*n* = 10). **d** Western blotting of midbrains isolated from mice was performed using antibodies to TH (left panel) and quantitative analysis of protein-band intensity (right panel). **e** TH-positive cells in the SNpc of mice exposed to Fen/vehicle via ST striatal injections stained by a TH antibody and visualized at ×4 and ×10 magnification (left panel); quantitative analysis of the number of TH-positive cells in the SNpc (right panel). **f** 4-HNE-positive cells in the SNpc of mice exposed to Fen/vehicle via ST striatal injections stained by a 4-HNE antibody; quantitative analysis of the number of 4-HNE-positive cells in the SNpc (right panel). For all quantitative/statistical analysis, *p < 0.05; **p < 0.01; and ***p < 0.001. All data are presented as means ± SEMs. Scale bars are 500 μm and 200 μm in **e** and 20 μm in **f**.

To investigate the toxic effects of Fen on DA neurons in mice, we administered single unilateral stereotaxic (ST) injections of Fen into the striatum to establish a model of unilateral injury. Compared to phenotypes in vehicle-treated mice, Fen induced restlessness and hyperexcitability during the initial 4 weeks following Fen injections, followed by a gradual onset of symptoms, including hypoexcitability, hypokinesia, and hair loss at ~6 weeks after Fen injections. At 24 weeks after the first Fen injection, mice exhibited back hunching and whole-body tremors. Behavioral assessments revealed that Fen-injected mice exhibited less distance traveled (Fig. 1a), less time spent on the rotarod (Fig. 1b), less grip strength (Fig. 1c), and weakened behavior in resisting arrest compared to these parameters in vehicle-treated mice, suggesting that chronic Fen treatment gradually reduced motor skills. Collectively, these behavioral results revealed impaired motor function after Fen exposure. Moreover, we found that the production of tyrosine hydroxylase (TH) and the number of TH-positive cells were significantly decreased in the SNpc of Fen-treated mice compared to these parameters in vehicle-treated mice (Fig. 1d, e), and TH expression was also reduced in the striatum of Fen-treated mice (Supplementary Fig. 1). Furthermore, an assessment in SNpc using immunohistochemical staining with a 4-HNE antibody—which recognizes oxidized proteins and lipids—showed that oxidized proteins and lipids were significantly increased in Fen-treated mice compared to these levels in vehicle-treated mice (Fig. 1f). Taken together, these results suggest that Fen induced oxidative stress that led to DA neurodegeneration and PD-like behavioral deficits in mice.

### Fen induces oxidative stress in primary neurons in vitro

As a first step toward elucidating the mechanisms of neuronal degeneration by Fen in vivo, primary cultured neurons from C57BL/6 mice were used in vitro and neuronal identity was determined via immunofluorescence staining with a microtubule associated protein 2 (MAP-2) antibody (Supplementary Fig. 2). Fen treatment reduced cellular viability in a concentration-dependent manner (Supplementary Fig. 3). About 40% of cells show low viability at 100 μM of Fen and this concentration was further used.

**Fig. 2:**
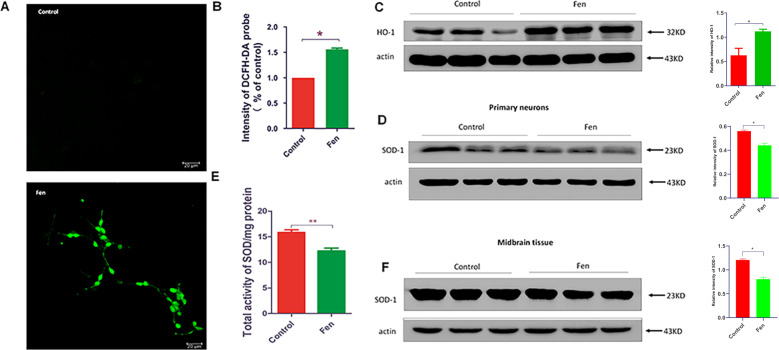
Fen induces oxidative stress in primary neurons in vitro. **a** Cultured primary neurons treated with Fen (100 μM) for 24 h, and intracellular ROS using the DCFH-DA probe was recorded by confocal microscopy. **b** Quantitative analysis of fluorescent intensity of the DCFH-DA probe by ELISA. **c**, **d** Western blotting of primary neuronal lysates was performed using antibodies to HO-1 and SOD-1 (left panel), and quantitative analysis of protein-band intensities are shown (right panel). **e** The activity of total SOD was measured by ELISA. **f** Western blotting of midbrain tissue lysates of mice was performed using antibodies to SOD-1 (left panel) and quantitative analysis of protein-band intensities are shown (right panel). For all quantitative/statistical analysis, *p < 0.05. All data are presented as means ± SEMs. Scale bars are 20 μm in **a**.

A previous study has indicated that oxidative stress originating from mitochondria may contribute to neurodegeneration^30^. Moreover, in our present study, we found that large amounts of oxidized proteins and lipids appeared in the SNpc of Fen-treated. Thus, we next measured intracellular ROS levels in Fen-treated primary neurons in vitro via a 2′,7′-dichlorodihydrofluorescein diacetate (DCFH)-DA probe. Fen significantly increased ROS levels by ~50% comparing to that of no treatment (Fig. 2a, b). Furthermore, Fen-evoked oxidative stress may induce activation of cytoprotective processes to counteract free-radical-mediated damage of cells and molecules. Hence, we next determined the activities of selected enzymes and protein expression levels involved in antioxidative defense against oxidative damage. Fen significantly increased the protein level of inducible heme oxygenase 1 (HO-1) (Fig. 2c) and decreased the expression of superoxide dismutase-1 (SOD-1) protein and total activity of (SOD) in vitro (Fig. 2d, e); additionally, Fen significantly decreased the expression of SOD-1 protein in vivo (Fig. 2f). Taken together, these data suggest that Fen induced higher levels of ROS and reduced antioxidant defense in primary neurons in vitro, and that some of these effects may have been recapitulated in vivo.

### Fen disrupts mitochondrial dynamics and mitochondrial morphology and reduces mitochondrial mass in vitro

**Fig. 3:**
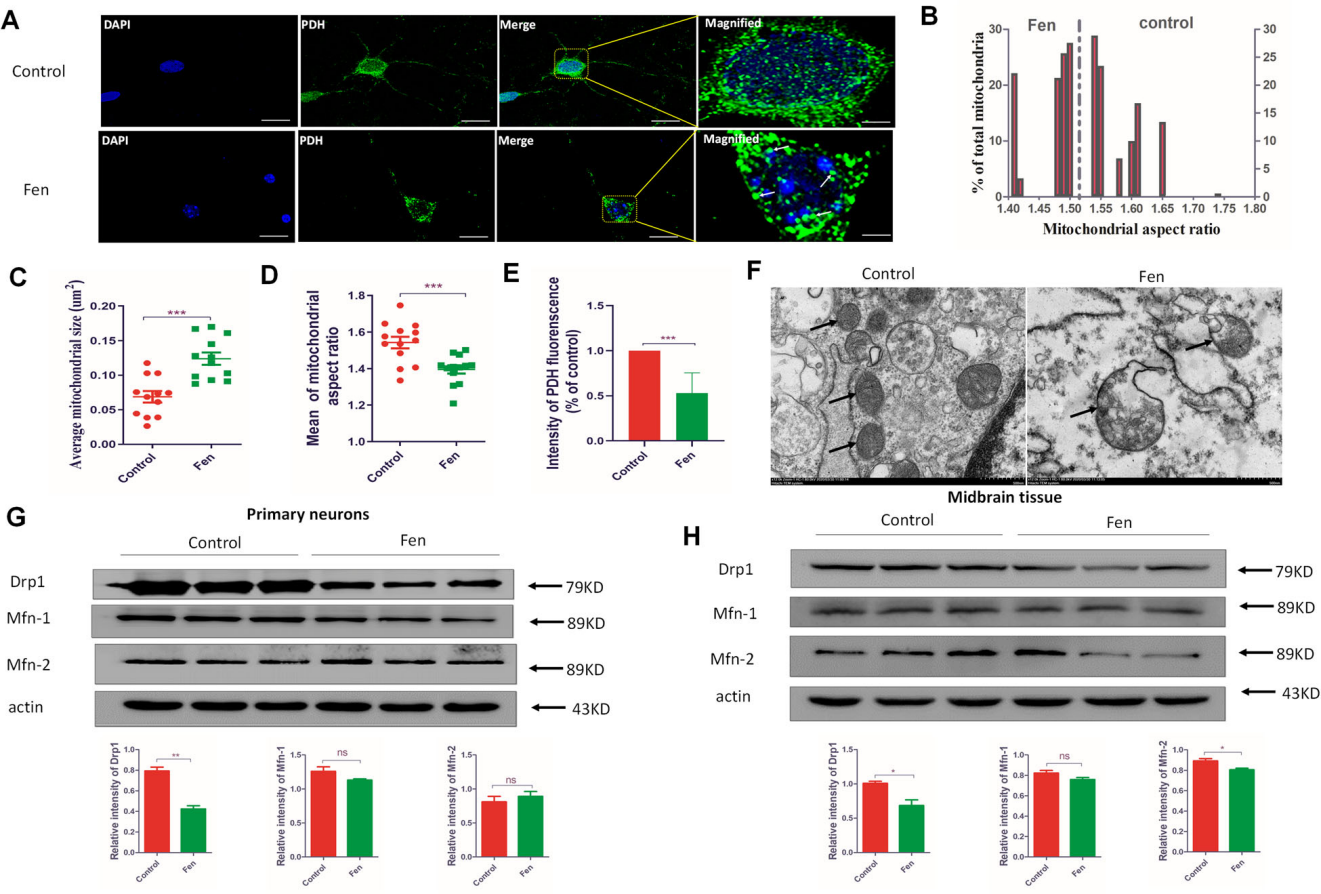
Fen disrupts mitochondrial dynamics and morphology and reduces mitochondrial mass in vitro. **a** Mitochondria were analyzed by confocal microscopy with anti-PDH antibodies (a mitochondrial matrix protein, in green) and DAPI. White arrows indicate swollen mitochondria. **b** Histogram of mitochondrial aspect ratios. **c** Average mitochondrial size (*n* = 4334 mitochondria from the control group, *n* = 709 mitochondria from with Fen group). **d** Average mitochondrial aspect ratio (*n* = 4334 mitochondria from the control group, *n* = 709 mitochondria from the Fen group). **e** Quantitative analysis of PDH fluorescence. **f** The represent image of TEM. Black arrows indicate swollen mitochondria. **g** In vitro and **h** in vivo protein levels of Drp1, Mfn-1, and Mfn-2 detected by Western blotting (above panel) and quantitative analysis of protein-band intensities (below panel). Figure 2f and **h** are represent same samples, therefore, Actin in Fig. 2f and **h** are completely identical. For all quantitative/statistical analysis, *p < 0.05; **p < 0.01; ***p < 0.001, and “ns” denotes no significant difference. All data are presented as means ± SEMs. Scale bars are 20 μm in **a** and 500 nm in **f**.

ROS is a by-production of energy generation derived from mitochondrial oxidative phosphorylation, and harmonious mitochondrial function is required to prevent excessive ROS generation. Healthy mitochondrial morphology and dynamics are essential for maintaining mitochondrial function. Therefore, further experiments were performed to determine Fen-induced changes in mitochondrial morphology and dynamics. Immunofluorescent experiments with anti-pyruvate dehydrogenase (PDH, a mitochondrial matrix protein) antibodies were performed to assess mitochondrial morphology. Fen changed the shape of mitochondria into large spheres within neurons (as indicated by the white arrows in Fig. 3a) and the mitochondrial aspect ratio was significantly left-shifted compared to that of controls (Fig. 3b); additionally, Fen significantly increased the average mitochondrial size and decreased the mitochondrial aspect ratio compared to these parameters in controls (Fig. 3c, d). Meanwhile, Fen also significantly increased the mitochondrial average length and width, equivalent spherical diameter, and average circularity (Supplementary Fig. 4a–d). Moreover, the relative fluorescent intensity of PDH in Fen-treated primary neurons was significantly reduced compared to that of controls (Fig. 3e); this result indicated that the mitochondrial mass was decreased. Subsequently, we observed the mitochondrial morphology using transmission electron microscopy (TEM). We also found that Fen significantly increased the size of mitochondria and impaired the mitochondrial morphology and the mitochondrial cristae disappeared (as indicated by the black arrows in Fig. 3f). Since mitochondrial dynamics are closely related to mitochondrial morphology, we next measured regulatory factors associated with mitochondrial dynamics. The results of immunoblotting experiments shown that the Fen-induced level of Drp1 expression involved in mitochondrial division was significantly reduced compared to that in controls both in vitro (Fig. 3g) and in vivo (Fig. 3h). However, the expression levels of Mfn-1 and Mfn-2—both of which are involved in mitochondrial fusion—were not affected by Fen treatment in vitro (Fig. 3g) or in vivo (Fig. 3h). In addition, the Fen-induced swollen mitochondria that we observed appeared similar to those in Drp1-KO neurons treated with hydrogen peroxide in a previous study^31^. Next, to determine whether the formation of spherical mitochondria in Fen-treated neurons was due to increased oxidative stress, we incubated primary neurons with an antioxidant, N-acetylcysteine (NAC). We found that NAC significantly increased the mitochondrial aspect ratio and reduced both the mean mitochondrial width and the average mitochondrial size in Fen-treated primary neurons, whereas the average mitochondrial length was unaffected (Supplementary Fig. 5a–e). These results demonstrate that Fen-induced oxidative stress may contribute to increased mitochondrial width rather than affecting mitochondrial length. Overall, these findings indicate that Fen perturbed mitochondrial dynamics/morphology and reduced mitochondrial mass in primary neurons in vitro.

### Fen disrupts mitochondrial function and impairs synaptic transmission in primary neurons

**Fig. 4:**
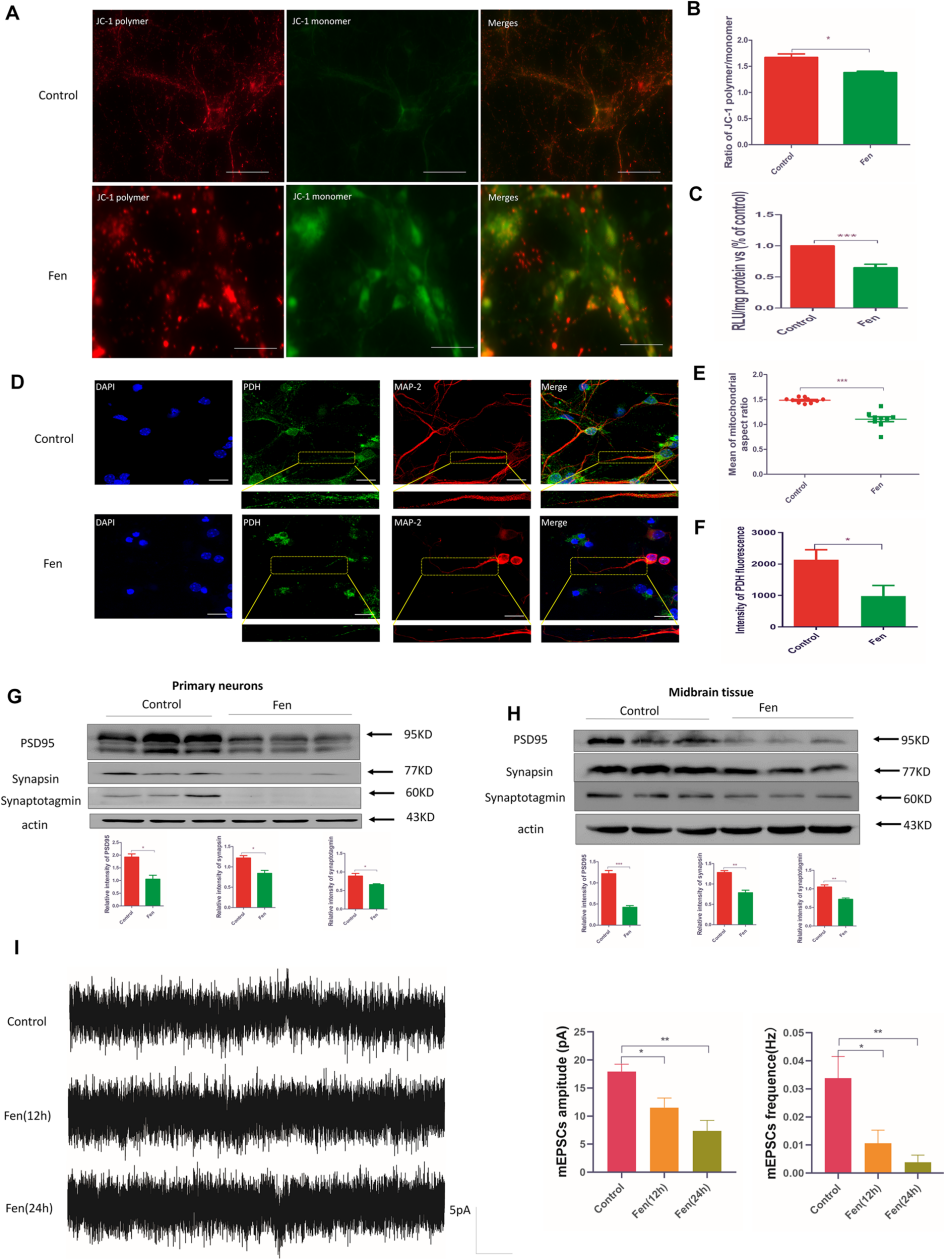
Fen disrupts mitochondrial function and impairs synaptic transmission in primary neurons in vitro. **a** The represent image of fluorescence microscope. The polymer statu of JC-1 indicate the higher MMP (red) and the monomer statu of JC-1 indicate the lower MMP (green). **b** Ratio of red fluorescence to green fluorescence. **c** ATP levels from neuronal lysates. **d** Confocal microscopy of cultured neurons using a PDH antibody (green) and Map-2 antibody (red). The boxed area in each subpanel is magnified below its corresponding subpanel and shows neurites. **e** Mean mitochondrial aspect ratio in the box areas in **c**. **f** Quantitative analysis of the green fluorescent intensity in the boxed area (1367.02 μm length of neurites from the control group, 1058.74 μm length of neurites from the Fen group). **g** In vivo and **h** in vitro protein levels of PSD-95, synaptotagmin, and synapsin detected by Western blotting (top panel) and quantitative analysis of protein-band intensities are shown (bottom panel). **i** Averaged mEPSCs from an example recording are shown (left panel), and quantitative analysis of frequency and amplitude are shown (right panel). Figure 1d and **h** are represent same samples, therefore, Actin in Fig. 1d and **h** are completely identical. For all quantitative/statistical analysis, *p < 0.05; **p < 0.01; and ***p < 0.001. All data are presented as means ± SEM. Scale bars are 20 μm in **c**.

Mitochondria are essential to cellular metabolism and physiology, especially in the process of energy metabolism. To analyze the effect of Fen on mitochondrial function, JC-1 staining was used to evaluate the mitochondrial membrane potential (MMP). Fen significantly decreased the MMP by ~30% compared to that of control cells (Fig. 4a, b). As the major organelle for ATP production, the concentration of ATP is closely related to mitochondrial function in cells. We found that the concentration of ATP was reduced in Fen-treated cells compared to that in control cells (Fig. 4c); this result suggests that Fen damaged mitochondrial respiratory function. A previous report indicated that Drp1-dependent division is required for efficient mitochondrial transport and for maintaining synaptic function^31–33^. Hence, Drp1 deficiency and reduced ATP production may decrease the capability of mitochondrial movement toward synaptic terminals and lead to mitochondrial depletion in axon terminals as subsequent neurodegeneration^32,34,35^. Given that we found decreased Drp1 expression in Fen-treated primary neurons, we next investigated mitochondrial morphology and mass in neurites. Following Fen treatment, we found that the remnant mitochondrial morphology was also abnormal (Fig. 4d, e) and that mitochondrial mass (assayed via anti-PDH fluorescence) was significantly reduced in neurites (Fig. 4f) compared to these parameters in controls. These results may be due to either Fen-induced reductions in Drp1 blocking delivery of mitochondria to axonal terminals, or disruption of mitochondrial morphology within neurites. Consistently, mitochondrial turnover and sufficient ATP production are required for maintaining synaptic communication and neuronal responses to stimuli^34,36,37^. Next, we measured the expression levels of synaptotagmin, synapsin, and postsynaptic density protein-95^38^, and found that their expression levels were significantly reduced both in vitro (Fig. 4g) and in vivo (Fig. 4h). Meanwhile, we recorded miniature excitatory postsynaptic currents (mEPSCs) in the whole-cell configuration via patch-clamp electrophysiological recordings. Consistent with our immunoblotting results, the mEPSC amplitudes and frequencies were significantly reduced in Fen-treated primary neurons compared with those of controls (Fig. 4i). Collectively, these results suggest that Fen disrupted mitochondrial function and synaptic transmission in primary neurons.

### Fen enhances autophagic flux and mitophagy

**Fig. 5:**
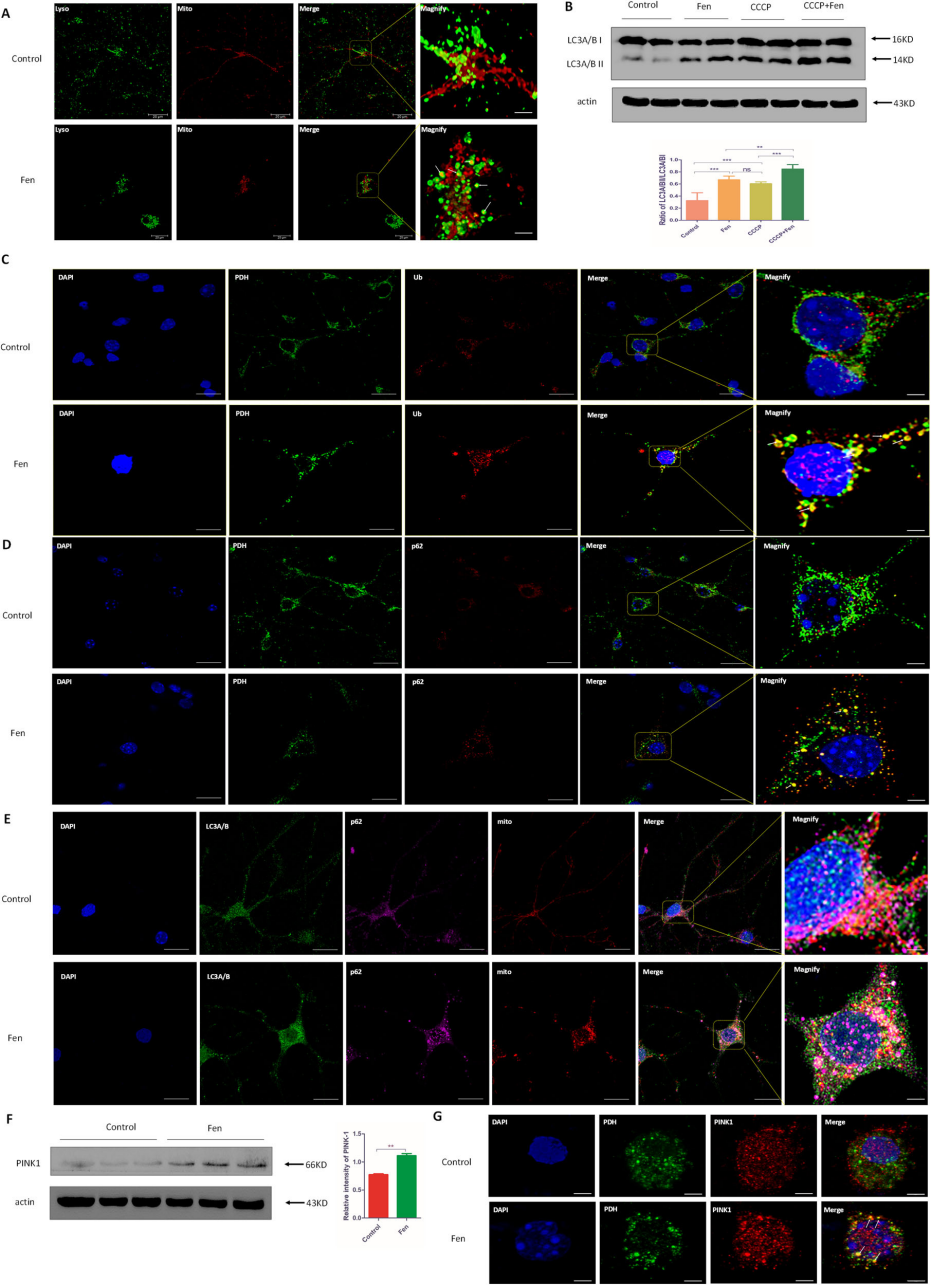
Fen enhances autophagic flux and mitophagy in primary neurons. **a** Representative images of primary neurons, with or without Fen treatment after 24 h, probed with Mitotracker (red) and Lysotracker (green). Arrows denote co-localization of mitochondria and lysosomes. **b** Western blotting of cultured neuronal lysates was performed using antibodies to LC3A/B (top panel) and quantitative analysis of the LC3A/B II/LC3A/B I ratio is shown (bottom panel); CCCP (10 µM) was used as an inducer of mitophagy. **c** Representative images of primary neurons, with or without Fen treatment after 24 h, stained with antibodies to PDH (green) and Ub (red). **d** Representative images of primary neurons, with or without Fen treatment over 24 h, stained with antibodies to PDH (green) and p62 (red). **e** Representative images of primary neurons, with or without Fen treatment over 24 h, stained with antibodies to LC3A/B (green), p62 (purple), and Mitotracker (red). **f** Western blotting of cultured neuronal lysates was performed using antibodies to PINK1 (left panel) and quantitative analysis of protein-band intensities is shown (right panel). **g** Representative images of primary neurons, with or without Fen treatment over 24 h, stained with antibodies to PDH (green) and PINK1 (red). For all quantitative/statistical analysis, *p < 0.05; **p < 0.01; and ***p < 0.001. All data are presented as means ± SEMs. Scale bars are 20 μm in **a**, **c**–**e**, and 3 μm in **g**.

Since damaged mitochondria pose a lethal threat to cells, prompt removal of damaged mitochondria is necessary for cellular survival. To investigate the capacity of damaged mitochondria being removed following Fen treatment, we next assayed mitophagy, which is a mitochondrial quality control system for eliminating damaged mitochondria^39^. Hence, we first assayed autophagic flux via measuring the expression levels of ubiquitin, p62, and LC3A/B—all of which are involved in the process of autophagy—and chloroquine (CQ), the latter of which is an inhibitor of autophagy. We found that autophagic intermediates, such as ubiquitin (Supplementary Fig. 6a) and p62 (Supplementary Fig. 6b), were significantly decreased via Fen treatment in vitro, and that the ratio of LC3A/B II/LC3A/B I was significantly increased following Fen treatment both in vitro (Supplementary Fig. 6c) and in vivo (Supplementary Fig. 6d). These results demonstrated that autophagic flux was increased in Fen-treated primary neurons and that this phenotype may have also been recapitulated in vivo. Earlier in our present study, we found that mitochondrial function and morphology were dysregulated via Fen treatment; as such, we next investigated whether Fen-induced alterations of autophagic intermediates were due to activation of mitophagy. Thus, we first detected the co-localization of mitochondria and lysosomes using a MitoTracker probe and LysoTracker probe, respectively^40^. We found that co-localization of mitochondria/lysosomes was significantly increased in Fen-treated cells compared to that of controls (Fig. 5a). This result supports the notion that the elimination of mitochondria was enhanced following Fen treatment. Subsequently, we used the chemical reagent, carbonyl cyanide m-chlorophenylhydrazone (CCCP), as an inducer of mitophagy and then detected the resultant ratio of LC3A/B II/LC3A/B I. Immunoblotting showed that Fen exerted a similar mitophagic effect to that of CCCP and, when used together, the effect of Fen/CCCP co-treatment was significantly greater than either treatment alone (Fig. 5b). These results demonstrated that Fen may have acted as an inducer of mitophagy. In addition, we investigated whether neurons accumulated mitophagic intermediates, as assessed via confocal microscopy with antibodies to PDH, p62, ubiquitin, and LC3A/B. We found that ubiquitin, p62, and LC3A/B accumulated around mitochondria in Fen-treated neurons, as compared with those in control neurons (Fig. 5c–e). And the image of TEM also indicated that Fen promoted the formation of autophagosome containing mitochondria (Supplementary Fig. 7). These results further indicated that mitophagy was enhanced in primary neurons treat with Fen in vitro. Since PINK1 plays an important role in the initiation of mitophagy, we next measured the expression of PINK1 via immunoblotting and used confocal microscopy to assess the PINK1 localization. PINK1 expression was significantly increased and was spatially localized to mitochondria following Fen treatment, confirming that Fen induced the onset of mitophagy (Fig. 5f, g). Taken together, these findings demonstrate that autophagic flux was enhanced in Fen-treated primary neurons, which may have been derived from enhanced mitophagy.

## Discussion

Although previous studies in cell lines and in rats have previously demonstrated Fen-induced neurodegeneration^5,29^, the underlying mechanisms of this phenomenon have remained unclear. In our present study, we focused on the effect of Fen on mitochondria, because considerable evidence has shown that mitochondria play a critical role in the progression of neurodegeneration. First, we administered ST injections of Fen into the striatum of mice and found that Fen induced degeneration of SNpc DA neurons and PD-like behavioral deficits, suggesting that Fen exposure may be a risk factor for PD. In addition, we next investigated the underlying mechanism of Fen-induced neurodegeneration in cultured primary neurons. We found that Fen disrupted mitochondrial function/dynamics and enhanced mitophagy. According to our present results and those of a previous study^41^, we conclude that Fen reduced mitochondrial mass and impaired mitochondrial function via disturbing mitochondrial dynamics, which ultimately disrupted synaptic transmission and induced neurodegeneration. A balance of mitochondrial dynamics may therefore represent a convergence between mitochondrial function and neuronal survival during exposure to Fen.

Oxidative stress (OS) and excess ROS production from mitochondria are considered to contribute to neuronal degeneration and are involved in the etiology of PD^15,42–45^. Previous studies have indicated that when neurons are hyperactive, they are more susceptible to OS and/or ROS in the brain. Long-term exposure of cells or tissues to OS or excessive ROS can lead to cellular and tissue damage. In accordance with previous reports^46^, our present study revealed that Fen significantly upregulated ROS levels and HO-1 expression and reduced both the expression of SOD-1 and the enzymatic activity of total SOD-1 in primary neurons. Correspondingly, the expression of SOD-1 was also significantly decreased, whereas oxidative proteins and lipids were increased, in the SNpc of striatal Fen-treated mice. These results indicate that Fen induced both primary neurons in vitro and brain tissue in mice in vivo to undergo OS and to exhibit reduced antioxidative capabilities.

ROS is a byproduct in the process of mitochondrial-mediated energy metabolism^47^. Thus, healthy mitochondria are crucial for regulating the production of ROS. Furthermore, mitochondrial morphology and dynamics are closely related to mitochondrial function^48,49^. Our present data showed that the expression of Drp1—which contributes to mitochondrial fission—was significantly decreased following Fen treatment; however, the expression levels of Mfn-1 and Mfn-2 (which regulate mitochondrial fusion) were unaffected following Fen treatment. Yamada et al.^21^ indicated that the coordinated expression patterns between fission factors and fusion elements are vital to cellular development and survival; therefore, dysregulation, of either component will lead to cellular/tissue damage. Even a partial decrease in either mitochondrial fission or fusion has been shown to impair neuronal viability^50^. The loss of Drp1 protein can lead to mitochondria becoming elongated or shaped as large spheres in several different neuronal lines in vitro and causes premature death in murine models in vivo^51–53^. Our present results showed Fen-induced disrupted mitochondrial dynamics and morphology, the latter of which was exhibited in the form of swollen mitochondria (as indicated by arrows in Fig. 3a). These swollen mitochondria looked similar to those in Drp1-KO neurons treated with hydrogen peroxide^31^, suggesting increased amounts of oxidative damage. Interestingly, we also found that mitochondrial mass was decreased in Fen-treated primary. Since a previous study indicated that Drp1 is required for formation of new mitochondria^48^, we hypothesize that Fen-mediated downregulation of Drp1 may be contribute to reduced mitochondria mass in primary neurons. These results suggest that Fen may disturb the balance of mitochondrial dynamics and reduce mitochondria mass via suppressing Drp1 expression, as well as perturbing mitochondrial morphology and increasing ROS levels in cultured neurons.

Previous studies have shown that healthy mitochondrial dynamics are required to maintain mitochondrial respiratory function and synaptic transmission. For instance, it has been shown that oxidative damage is increased and mitochondrial respiratory activities are decreased following Drp1 knockdown in HeLa cells^41,54^. These results may be due to aberrant spherical mitochondrial morphology destroying the surface-area-to-volume ratio of mitochondria and disturbing the intra-mitochondrial distributions of mitochondrial DNA and matrix proteins, all of which likely perturb electron transport chain complexes and respiratory function^52^. Another study has indicated that Pyrs are potent inhibitors of mitochondrial complex I^20^. Our present results showed that the MMP and the production of ATP were decreased following Fen treatment, which suggests that Fen impaired mitochondrial function and inhibited mitochondrial respiratory chain function via disturbing mitochondrial dynamics and/or inhibiting mitochondrial complex I. In neurons, mitochondria are not only distributed in the soma, but are also transported along neurites and into axonal terminals, which require large amounts of energy^55^. Mitochondrial fission is critical for proper synaptic function, as the disruption in the regulation of mitochondrial dynamics may be involved in altered synaptic mitochondrial mass and turnover, which may ultimately lead to neurodegeneration due to insufficient energy^36,56^. Previous studies have also indicated that DA neurons are susceptible to toxicity induced via Drp1 deficiency^32^. The mitochondrial mass has been shown to be significantly decreased in axons of Drp1-KO DA neurons, which leads to the degradation of synapses prior to that of the soma^32^. The degradation of synapses and degeneration of cells occurring in Drp1-KO DA neurons or Purkinje cells has been shown to be due to suppression of mitochondrial mobility and respiratory function^31,32,52^. Consistent with these previous findings, our present study showed that mitochondrial mass was decreased in the neurites of Fen-treated primary neurons. Meanwhile, we also observed that mitochondrial morphology in neurites was abnormal in Fen-treated primary neurons. This result may have been due to Fen inhibiting the expression of Drp1, leading to a decrease in mitochondrial fission, increased mitochondrial content, and/or disruption of normal mitochondrial movement in the form of unusual asymmetric extension–retraction movements. Fen may have also decreased ATP production due to reduced mitochondrial mass or disturbed mitochondrial function, which result from the enhanced elimination or decreased regeneration of mitochondria mediated by Drp1 in Fen-treated primary neurons.

Under physiological conditions, mitochondrial elimination and replenishment reach an equilibrium to allow for the maintenance of mitochondrial volume. Mitochondrial removal is mainly performed via mitophagy. However, the dysregulation of mitophagy—including inhibited mitophagy and excessive mitophagy—is considered to contribute to PD. Numerous studies have shown that inhibited mitophagy leads to the accumulation of dysfunctional mitochondria^57^. On the contrary, excessive mitophagy induces a decrease in mitochondria mass and insufficient ATP production^58^. Hence, we hypothesized that the decreased mitochondrial mass in Fen-treated cells may have been partially due to enhanced mitophagy. To test this hypothesis, we first confirmed that Fen enhanced autophagic flux, as shown by the formation of autophagosomes, increased autophagic hallmarks (e.g., LC3A/B II/LC3A/B I ratio), and decreased expression levels of Ub and p62/SQSTM1. These results are in accordance with a previous study that found that natural Prys can induce autophagy in HepG2 cells^28^. Based on the observed Fen-induced mitochondrial dysfunction (Fig. 4a–c), we speculated that upregulated mitophagy may have likely contributed to enhanced autophagy. In order to test this hypothesis, we detected the progression of mitophagy using Western blotting and immunofluorescence. We found increased co-localization of mitochondria/lysosomes and increased accumulated mitophagy-related proteins (e.g., Ub, p62/SQSTM1, and LC3A/B) following Fen treatment, all of which indicated that Fen enhanced mitochondrial elimination^7,21,25^. Numerous studies have shown that PINK1/Parkin mutations are associated with familial recessive early-onset PD and play an essential role in PINK1/Parkin-dependent mitophagy^12^. Under physiological states, the expression of PINK1 is rarely detected because these proteins are imported into mitochondria and degraded by PINK1/PGAM5-associated rhomboid-like protease; however, PINK1 can be detected during enhanced PINK1/Parkin-dependent mitophagy because the import of PINK1 is blocked and accumulated on the outer mitochondrial membrane^59^. In our current study, we also observed that the expression of PINK1 was significantly increased and was preferentially wrapped around mitochondria following Fen treatment. Hence, we conclude that Fen may boost mitophagy via PINK1/Parkin-dependent pathways.

**Fig. 6:**
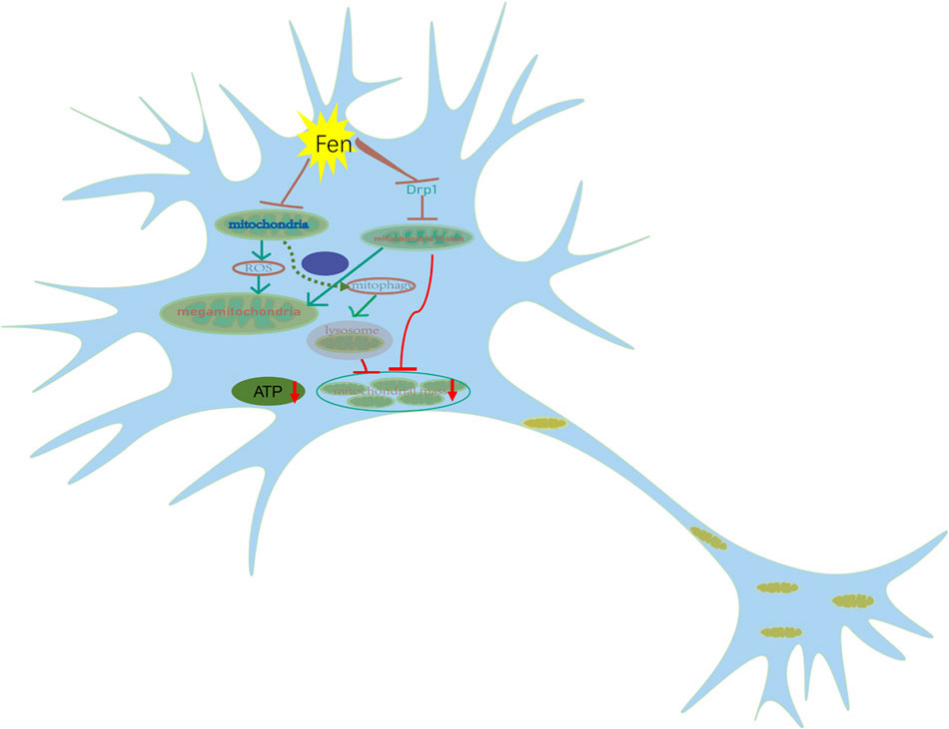
Hypothetical mechanism of Fen-induced neuronal degeneration. Fen induces the generation of mega-mitochondria via increasing the production of ROS and decreasing mitochondrial fission through suppressing the expression of Drp1. Meanwhile, Fen-induced decreases in Drp1 may lead to a reduction in mitochondrial regeneration and enhanced mitophagy, both of which may result in reduced mitochondrial mass and lower ATP levels. Fen-induced dysregulation of mitochondrial mass and reduced ATP levels may ultimately lead to neurodegeneration.

Collectively, our present study provides evidence that Fen disrupted mitochondrial dynamics and function and promoted mitophagy, all of which may have contributed to the degeneration of neurons in vitro and in vivo (Fig. 6). Therefore, Fen-induced disruption of the mitochondrial quality control system may contribute to neurodegeneration. In order to further elucidate the mechanisms of Fen-induced neurodegeneration, future studies should investigate other components of mitochondrial dynamics and mitophagic pathways to better determine the Fen-induced interaction between mitochondrial dynamics and mitophagy.

## Materials and methods

### Antibodies and reagents

The following antibodies and chemicals were used in this study: TH mouse monoclonal antibody (Santa Cruz Biotechnology, sc-25269), HO-1 mouse antibody (ProteinTech, 10701-1-AP), SOD-1 mouse monoclonal antibody (Santa Cruz Biotechnology, sc-101523), Drp1 rabbit monoclonal antibody (EnoGene, E2A-7037), Mfn-1 and Mfn-2 rabbit polyclonal antibody (BOSTER Biotechnology, PB0263 and BA1790-2), PDH mouse monoclonal antibody (Santa Cruz Biotechnology, sc-377092), p62/SQSTM1 rabbit polyclonal antibody (Cell Signaling Technology, #23214), LC3A/B rabbit polyclonal antibody (Cell Signaling Technology, #12741), PINK1 rabbit polyclonal antibody (Affinity Biotechnology, DF7742), MitoTracker (ThermoFisher Scientific, M22425), LysoTracker (ThermoFisher Scientific, L7528), PSD-95 rabbit polyclonal antibody (Cell Signaling Technology, #3450), synaptotagmin rabbit polyclonal antibody (Cell Signaling Technology, #14558), synapsin mouse monoclonal antibody (Santa Cruz Biotechnology, sc-136086), 4-HNE Monoclonal Mouse IgG_2B_ antibody (R&D Systems, MAB3249), actin Monoclonal Mouse antibody (Beyotime Biotechnology, AA128), horseradish peroxidase-conjugated goat anti-mouse (Beyotime Biotechnology, A0216), horseradish peroxidase-conjugated goat anti-rabbit (Beyotime Biotechnology, A0208), anti-Alexa Fluor 488-conjugated goat anti-mouse (BOSTER Biotechnology, BA1126), anti-Alexa Fluor 555-conjugated goat anti-rabbit (Boster Biotechnology, BA1135), reactive oxygen species assay kit (Beyotime Biotechnology, S0033), mitochondrial membrane potential assay kit with JC-1 (Beyotime Biotechnology, C2006), enhanced ATP Assay Kit (Beyotime, S0027), total superoxide dismutase assay kit with WST-8 (Beyotime, S010), fenpropathrin (Dr. Ehrensorfer, C13530000), NAC (Beyotime, S0077), CCCP (Solarbio Life Sciences, C6700), CQ (Sigma-Aldrich, C6628), and Restore-Plus Western-Blot stripping Buffer (ThermoFisher Scientific, 46430).

### Animals and treatments

Adult male C57BL/6 mice (22–25 g) were obtained from the Medical Experimental Animal Center of Guangdong (Foshan, China). Mice were housed in cages with under a 12-h light/dark cycle with access to food and water ad libitum. All animal protocols adhered to the Guide for the Care and Use of Laboratory Animals and were approved by the Institutional Animal Care and Use Committee of Southern Medical University (Guangzhou, China).

Animals were randomly divided into two groups: a DMSO-treated control group, and a Fen-treated group. DMSO was used to dissolve Fen. Based on a previous study^60^, we treated mice with a dose of 6.1 µg/g of brain weight. At 24 weeks after injections, the mice were euthanized and the midbrains were quickly dissected out and stored at −80 °C.

### Fenpropathrin-induced lesions

Mice were anesthetized with xylazine (5 mg/kg of body weight) and fixed on a ST instrument (RWD Life Science, Shenzhen, China) in a flat position. Mice received unilateral injections of Fen (1.092 mg/ml) or DMSO in 1 µl volumes (injection speed of 0.2 µl/min) into the right side of the striatum at the following coordinates: anterior 0.9 mm, lateral −2.0 mm, and ventral −3.0 mm. After the injection, the needle was left in place for 5 min to allow for complete diffusion of the drug before being slowly withdrawn from the brain. Mice were placed on a warming plate until they awoke and were then returned to their home cages.

### Open field test

The open field test consisted of a square arena (50 cm × 50 cm) with a white floor and 40-cm-high walls. The arena was brightly illuminated and had a central zone (25 cm × 25 cm) and a peripheral zone. Each mouse was gently placed in the center of the apparatus and observed for 10 min. The behavioral parameters (total distance) was recorded with a video camera and analyzed with Any-Maze behavioral tracking software (Ugo Basile, USA). After each trial, the apparatus was cleaned with 75% ethanol.

### Rotarod test

An accelerating rotarod test (Ugo Basile, USA) was performed as reported previously^61^ to evaluate motor coordination of mice. Briefly, the mice undertook a three-consecutive-day training period, consisting of three trials of 5 min each per day. This training method assures that all mice receive the same training time. Each mouse was tested three times in one experiment with 5-min resting intervals between tests. The rotarod was set to accelerate from 10 rpm to 40 rpm over a 5-min period. Each mouse was scored, in seconds, for the period of time it was able to stay on the rotating rod.

### Traction test

The traction test was used to assessed limb-movement coordination, as described in both our recent work and in another study^62,63^. A stainless steel bar (diameter of 1.5 mm, length of 30 cm) was fixed 30 cm over the base. Each mouse was hung by its forelimbs and left on the bar. The latency until each mouse fell down was recorded. Each mouse was assessed three times, with an interval of 5 min between each trial. For scoring in the traction test, the following criteria were employed: 0–4 s = 0; 5–9 s = 1; 10–14 s = 2; 15–19 s = 3; 20–24 s = 4; 25–29 s = 5; and over 30 s = 6.

### Primary neuronal cultures and treatments

Primary neuronal cultures were established and performed as described previously^38^. Primary neurons were derived from the cortices of C57BL/6 mice E16–18 pups. Briefly, the cortices were dissociated and the cells were collected following trypsinization and centrifugation. Dissociated cells were then plated on poly-l-lysine (0.33 mg/ml)-coated glass coverslips at a density of 4 × 10^5^ cells/cm^2^ in a 6-well plate and were maintained in Neurobasal A medium supplemented with B27, 1% penicillin/streptomycin, and ultraglutamine at 37 °C under 5% CO2 air in an incubator. Glial growth was inhibited by adding cytosine β-d-arabinofuranoside (10 μM) at 48 h after plating. Cells were grown for 7 days in vitro (DIV), ensuring half the medium was changed every 2 days. The presence of cortical neurons was determined by immunostaining with anti-MAP-2 expression. The cultured neurons were treated with 100 μM of Fen for 24 h.

### Cell counting kit-8 (CCK-8) assay

A total of 5 × 10^4^ primary neurons were seeded per well in 96-well plates. After 7 days in culture, cells were incubated with Fen (0, 25, 50, 75, 100, 125, 150, 175, or 200 μM) for 24 h. Cellular viability was measured using 2-(2-methoxy-4-nitrophenyl)-3-(4-nitrophenyl)-5-(2,4-disulfophenyl)-2H-tetrazolium monosodium salt (WST-8) with a CCK-8 assay (EnoGene Biotech Co., Ltd., Nanjing, China), according to the manufacturer’s instructions. The absorbance was determined at 450 nm using a multimode plate reader (PerkinElmer Inc., Hopkinton, MA, USA).

### Measurement of intracellular reactive oxygen species (ROS)

Intracellular ROS levels were determined using a DCFH-DA fluorescence assay (Beyotime). Briefly, neurons in confocal dishes were incubated with control media or Fen (100 μM) for 24 h. Treated neurons were then washed three times with sterilized phosphate-buffered saline (PBS, PH = 7.4) and incubated with DCFH-DA at 37 °C for 20 min. Subsequently, the neurons were gently washed three times with PBS to remove any remaining DCFH-DA. Images were acquired using laser-scanning confocal microscopy (Leica, Germany) with the same settings for all samples in each experiment. Fluorescent intensities were calculated using Leica LASX software (Leica, Germany).

### Intracellular superoxide dismutase (SOD) activity assay

Total SOD activity in primary neurons was determined using a Total SOD Assay Kit with WST-8 (Beyotime), according to the protocols provided by the manufacturer. Briefly, the cultured neurons were washed once in ice-cold PBS and buffer was added prior to centrifugation. The supernatant was collected and used for the analysis of both SOD activity and protein levels. The concentration of total protein was quantified via an Enhanced BCA Protein Assay Kit (Beyotime). The SOD activities were calculated by the ratio of total enzyme activity to the total protein level, and the results were expressed as U mg/protein.

### Intracellular ATP assay

ATP content was determined via an Enhanced ATP Assay Kit (Beyotime) according to the manufacturer’s instructions. The concentration of ATP was calculated according to the measured protein concentration and was expressed as RFU/mg of protein.

### Total protein extraction

Total protein extraction was performed as described previously^38^. Briefly, for total protein extraction from cultured neurons, the culture medium was discarded, cells were washed twice with ice-cold PBS, and were then harvested. For total protein extraction from midbrain samples, tissues were homogenized with a glass homogenizer, and cells or tissue homogenate were then centrifuged at 13,000 rpm for 3 min. Radioimmunoprecipitation assay buffer (Beyotime: contains 50 mM Tris (pH 7.4), 150 mM NaCl, 1% Triton X-100, 1% sodium deoxycholate, 0.1% SDS, sodium orthovanadate, sodium fluoride, EDTA, leupeptin) containing 1 mM phenylmethanesulfonyl fluoride was added, and the mixture was then placed on ice for 30 min. After centrifugation at 14,000 rpm for 15 min, the supernatant (containing the total protein fraction) was collected. The concentration of total protein was quantified via an Enhanced BCA Protein Assay Kit (Beyotime).

### Western blotting

Protein levels were determined via gel electrophoresis (12% or 15% SDS-PAGE) and probing using relevant antibodies. Peroxidase activity was detected by enhanced chemiluminescence (Beyotime), and chemiluminescent immunoreactive complexes were collected using the Bio-rad ChemiDoc MP imaging system (BIO-RAD). Quantitative analysis of protein-band intensities was executed using ImageJ software. Actin immunoreactivity was set as the internal control.

### Immunohistochemistry

Immunohistochemistry was performed as in our previous study^64^. Briefly, sections were cut into 10-μm slices and antigen retrieval was performed using citrate buffer. Sections were treated with 3% hydrogen peroxide (Sangon Biotech Co., Ltd., Shanghai, China) in PBS for 10 min and were then incubated in 5% BSA for 60 min. Sections were incubated overnight at 4 °C with primary antibodies as follows: TH (F-11) antibody (1:50; Santa Cruz Biotechnology Inc.), and 4-HNE antibody (1:50; R&D Systems.). After washing three times with PBS for 5 min each wash, sections were incubated sequentially in HRP-conjugated goat anti-mouse secondary antibody (ZSGB-BIO, PV6000, Beijing, China) for 1 h at room temperature. Sections were visualized with a 3,3-diaminobenzidine peroxidase substrate kit (ZSGB-BIO, Beijing, China). Integrated optical density was determined using an Image-Pro Plus 6.0 photogram analysis system (IPP 6.0; Media Cybernetics, Bethesda, MD, USA).

### Immunofluorescent assay

An immunofluorescent assay was performed as described previously^64^. Primary neurons that were grown in confocal dishes or on coated glass slides were incubated with primary antibodies overnight at 4 °C, rinsed with PBS, and incubated with Alexa Fluor 488-conjugated goat anti-mouse or Alexa Fluor 555-conjugated goat anti-rabbit IgG for 1 h at 37 °C. DAPI was used to stain cellular nuclei. Immunostaining was then examined using a Leica SP8 laser-scanning confocal microscope (Leica, Germany). Analysis of fluorescent intensities and particle features were performed by Leica LASX software (Leica, Germany).

### Whole‐cell patch‐clamp recordings

For whole-cell patch‐clamp recordings, culture medium was replaced with a bath solution containing the following (in mM): 140 NaCl, 3 KCl, 1.5 MgCl2, 10 HEPES, 11 Glucose, and 2.5 CaCl2 (pH = 7.4), which were supplemented with 5 μM tetrodotoxin and 2 μM bicuculline. The microelectrodes (3–6 MΩ) were tip‐filled with internal solution composed of 128 mM Cs‐methanesulfonate, 17.5 mM CsCl, 9 mM NaCl, 1 mM MgCl2, 0.2 mM EGTA, and 10 mM HEPES (pH 7.4). Cells were held at −70 mV in voltage‐clamp mode to record mEPSCs. Input and series resistances were monitored continuously, and data were discarded if either of these parameters changed by more than 20%. All data were obtained using a MultiClamp 700B patch‐clamp amplifier, sampled at 10 kHz, and filtered at 2 kHz using a Digidata 1550B analog–digital interface (molecular devices). mEPSCs were analyzed using Clamp fit 10.6 (Axon Instruments).

### Transmission electron microscopy (TEM)

Cultured neurons were washed three times with sterilized PBS (PH = 7.4) and were then gently scraped off with a cell scraper. Cells were collected into 1.5 ml Eppendorf tubes and centrifuged at 2000 rpm for 2 min. Supernatants were then discarded, and cellular pellets were fixed in 2.5% glutaraldehyde overnight, followed by 1% osmium tetroxide for 2 h. After dehydration and infiltration, cells were embedded in Spi-pon812 resin, polymerized, and then sectioned with a microtome (Leica EM UC7). Ultrathin sections were collected on copper grids, stained with uranyl acetate and lead citrate, and examined by TEM (Hitachi HT7800 120kv). Analysis was performed on digital images obtained from a CCD camera. Quantification of mitochondrial size was performed via ImageJ (NIH, Bethesda, MD, USA) by an examiner blind to the genotype and treatment of each sample.

### Statistics

Statistical analysis was performed via SPSS 22.0 (IBM Inc., Chicago, IL) using Student’s *t* tests for comparisons between two groups and one-way analyses of variance followed by Bonferroni post hoc tests for multiple comparisons. All data are expressed as the mean ± standard error of the mean, and the statistical significance level was considered as *p* < 0.05. Each analyzed dataset was derived from at least three independent experiments.

## Supplementary information

Supplementary Information

Supplementary Figure 1

Supplementary Figure 2

Supplementary Figure 3

Supplementary Figure 4

Supplementary Figure 5

Supplementary Figure 6

Supplementary Figure 7
