# Supplementary material for: Fenpropathrin induces degeneration of dopaminergic neurons via disruption of the mitochondrial quality control system
Source: Cell Death Discov. 2020 Aug 25;6:78. doi: 10.1038/s41420-020-00313-y (PMC7447795; doi:10.1038/s41420-020-00313-y)
Supplement: Supplementary file 1 — Supplementary Information [file 41420_2020_313_MOESM1_ESM.docx]

**Supplementary Information**

**Supplementary Figure legends**

**Supplementary Figure 1** Immunohistochemical staining of TH in the striatum of mice. TH-positive cells in the striatum of mice exposed to Fen via striatal ST injections, as stained by a TH antibody visualized at 4× and 10× magnifications. Scale bars are 500 μm (left) and 200 μm (right).

**Supplementary Figure 2** Immunofluorescent staining of MAP-2 in primary neurons *in vitro*. Primary neurons were cultured as described in the Material and Methods, and the neurons were stained with a MAP-2 antibody. DAPI was used to stain cellular nuclei. Scale bars are 20 μm.

**Supplementary Figure 3 Fen decreases cellular viability in primary neurons** *in vitro*. Cellular viability was assayed as described in the Materials and Methods.

**Supplementary Figure 4 Fen disrupts mitochondrial morphology in primary neurons** *in vitro*. (A) Average mean mitochondrial length (n = 4,334 mitochondria from the control group, n = 709 mitochondria from the Fen group). (B) Average mean mitochondrial width (n = 4,334 mitochondria from the control group, n = 709 mitochondria from the Fen group). (C) Average mitochondrial equivalent spherical diameter (n = 4,334 mitochondria from the control group, n = 709 mitochondria from the Fen group). (D) Average mean mitochondrial circularity (n = 4,334 mitochondria from the control group, n = 709 mitochondria from the Fen group). For all quantitative/statistical analysis, **p < 0.01 and ***p < 0.001.

**Supplementary Figure 5 NAC alleviates Fen-induced mitochondrial swelling in primary neurons** *in vitro***.** (A) Representative images of primary neurons, with or without Fen and Nac treatment over 24 h, stained with antibodies to PDH (green) and Map-2 (red). (B) Average mean mitochondrial aspect ratio. (C) Average mitochondrial width. (D) Average mitochondrial size. (E) Average mitochondrial length. Scale bars are 20 μm in (A). For all quantitative/statistical analysis, **p < 0.01 and ***p < 0.001.

**Supplementary** **Figure 6 Fen enhances autophagic flux both *in vitro* and *in vivo*.** (A) Western blotting of cultured neuronal lysates was performed using an antibody to Ub. (B) Western blotting of cultured neuronal lysates was performed using an antibody to p62 (left panel) and quantitative analysis of protein-band intensities is shown (right panel). (C) Western blotting of cultured neuronal lysates was performed using antibodies to LC3A/B (left panel) and quantitative analysis of the LC3A/B II/LC3A/B I ratio is shown (right panel); and CQ (25 µM) was used as an inhibitor of autophagy. (D) Western blotting of tissue lysates was performed using antibodies to LC3A/B (left panel) and quantitative analysis of the LC3A/B II/LC3A/B I is shown (right panel).

**Supplementary Figure 7. Fen enhances the formation of autophagosome containing mitochondria *in vitro*. (TIF, 6.08MB)**

The represent image of TEM. Black arrows indicate that the formation of autophagosome containing mitochondria. Scale bars are 500 nm.
