# Supplementary figures and images for: Fenpropathrin induces degeneration of dopaminergic neurons via disruption of the mitochondrial quality control system
Source: Cell Death Discov. 2020 Aug 25;6:78. doi: 10.1038/s41420-020-00313-y (PMC7447795; doi:10.1038/s41420-020-00313-y)

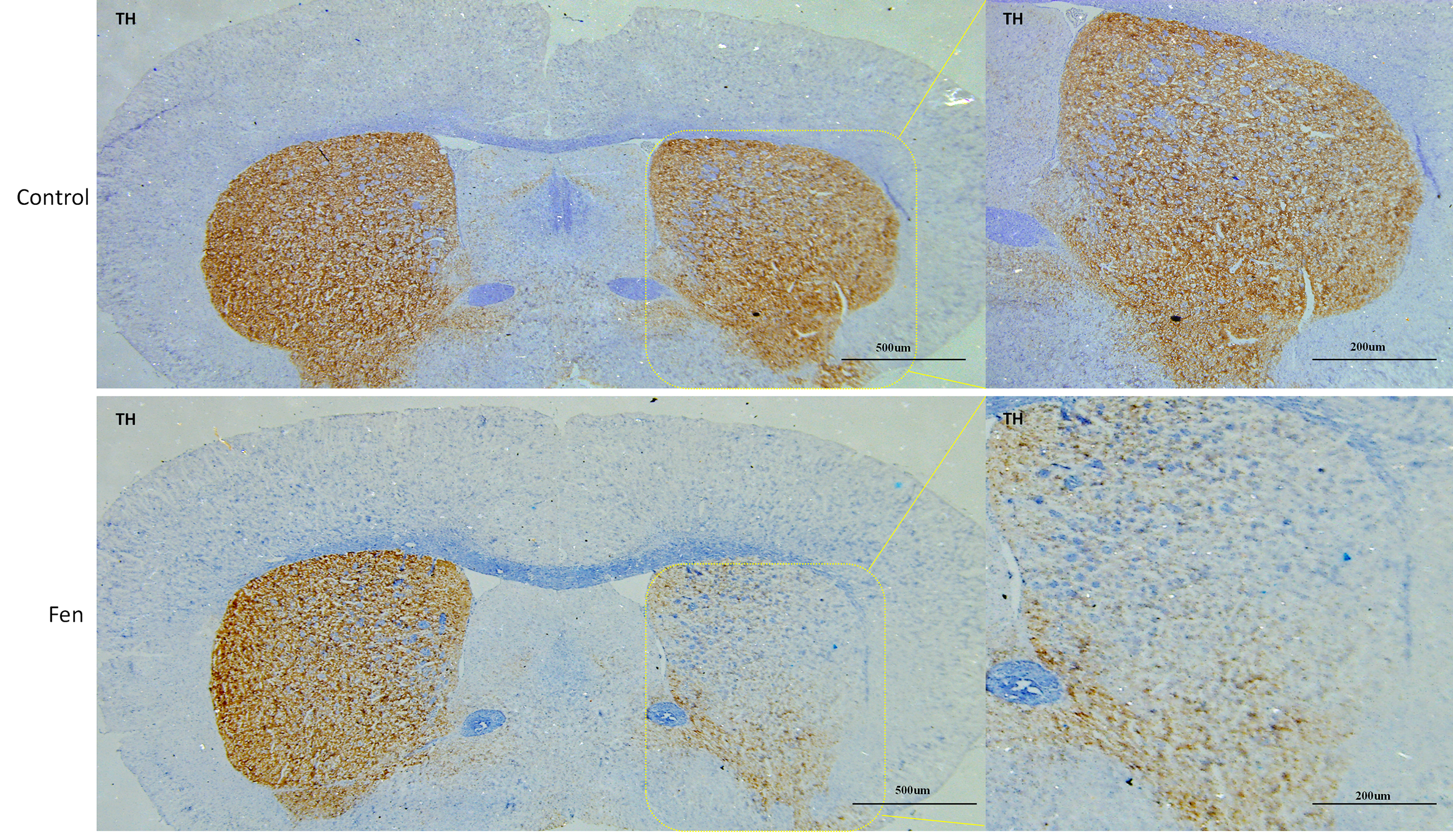

Supplement: Supplementary file 2 — Supplementary Figure 1 [file 41420_2020_313_MOESM2_ESM.tif]

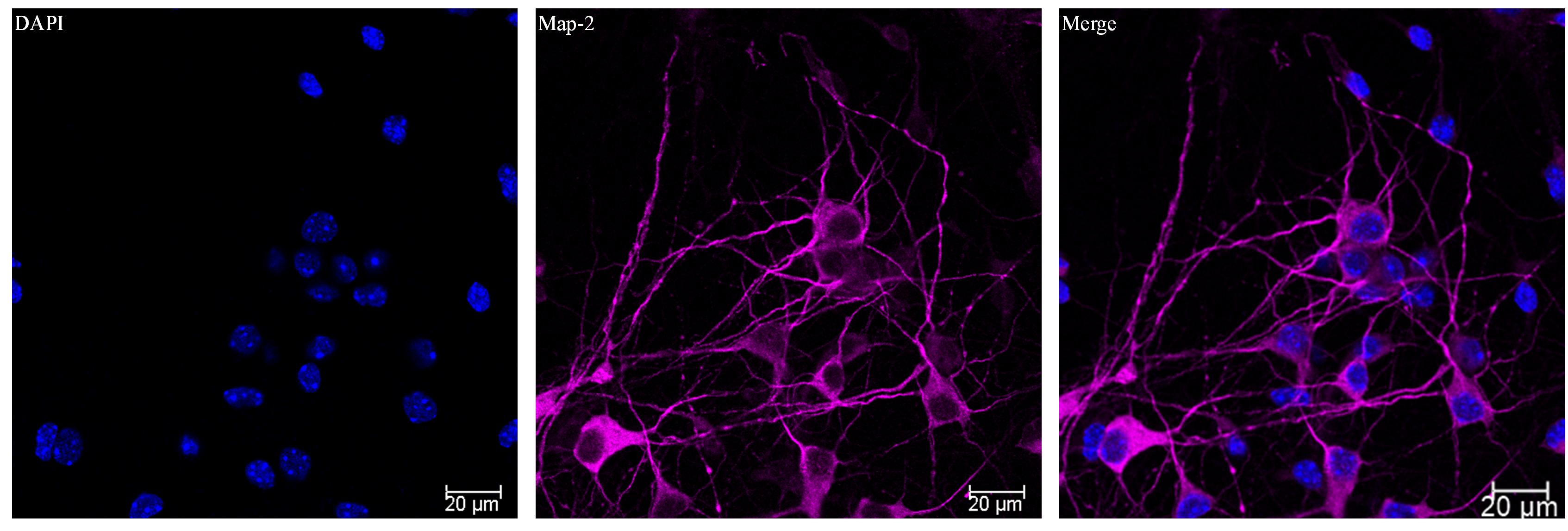

Supplement: Supplementary file 3 — Supplementary Figure 2 [file 41420_2020_313_MOESM3_ESM.tif]

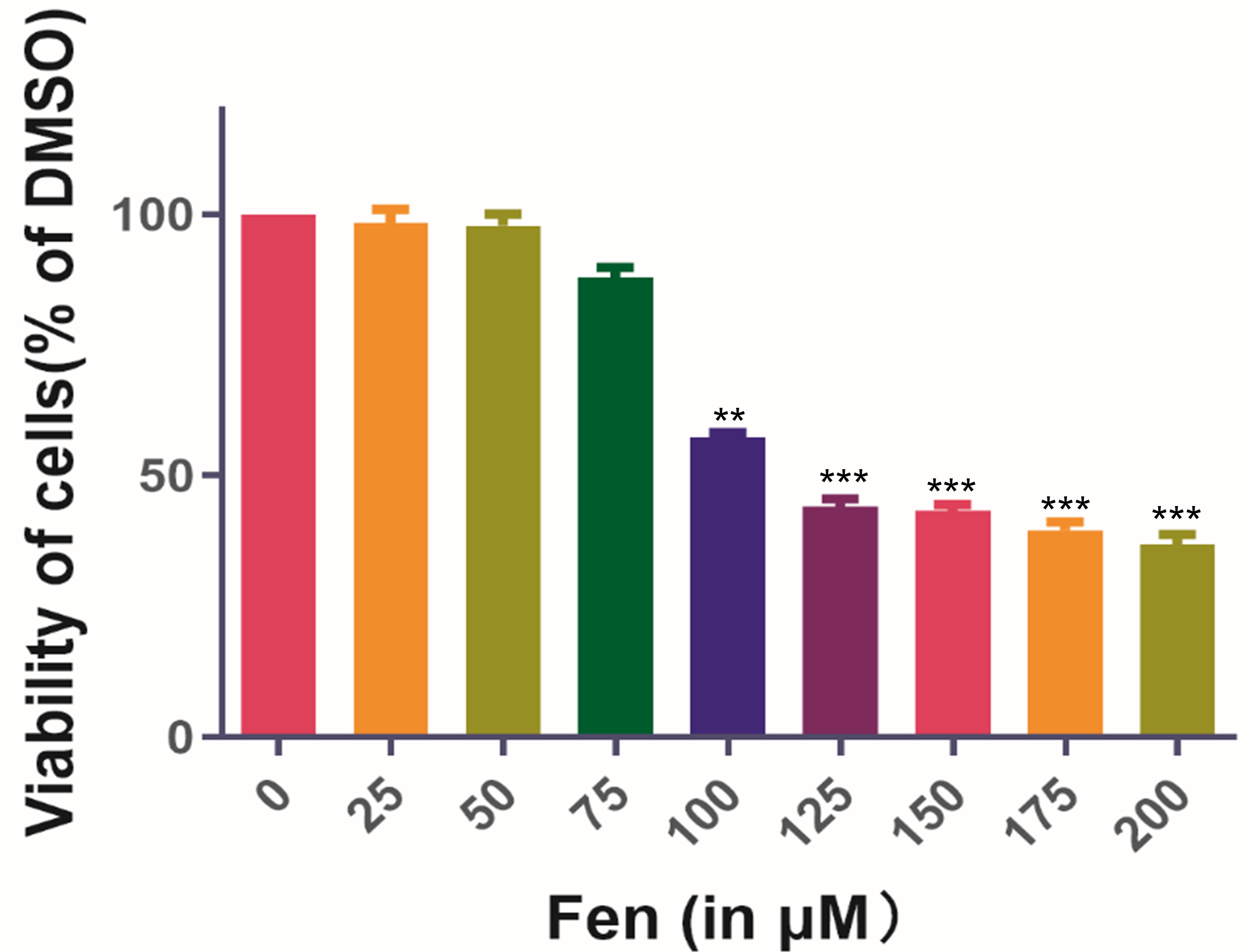

Supplement: Supplementary file 4 — Supplementary Figure 3 [file 41420_2020_313_MOESM4_ESM.tif]

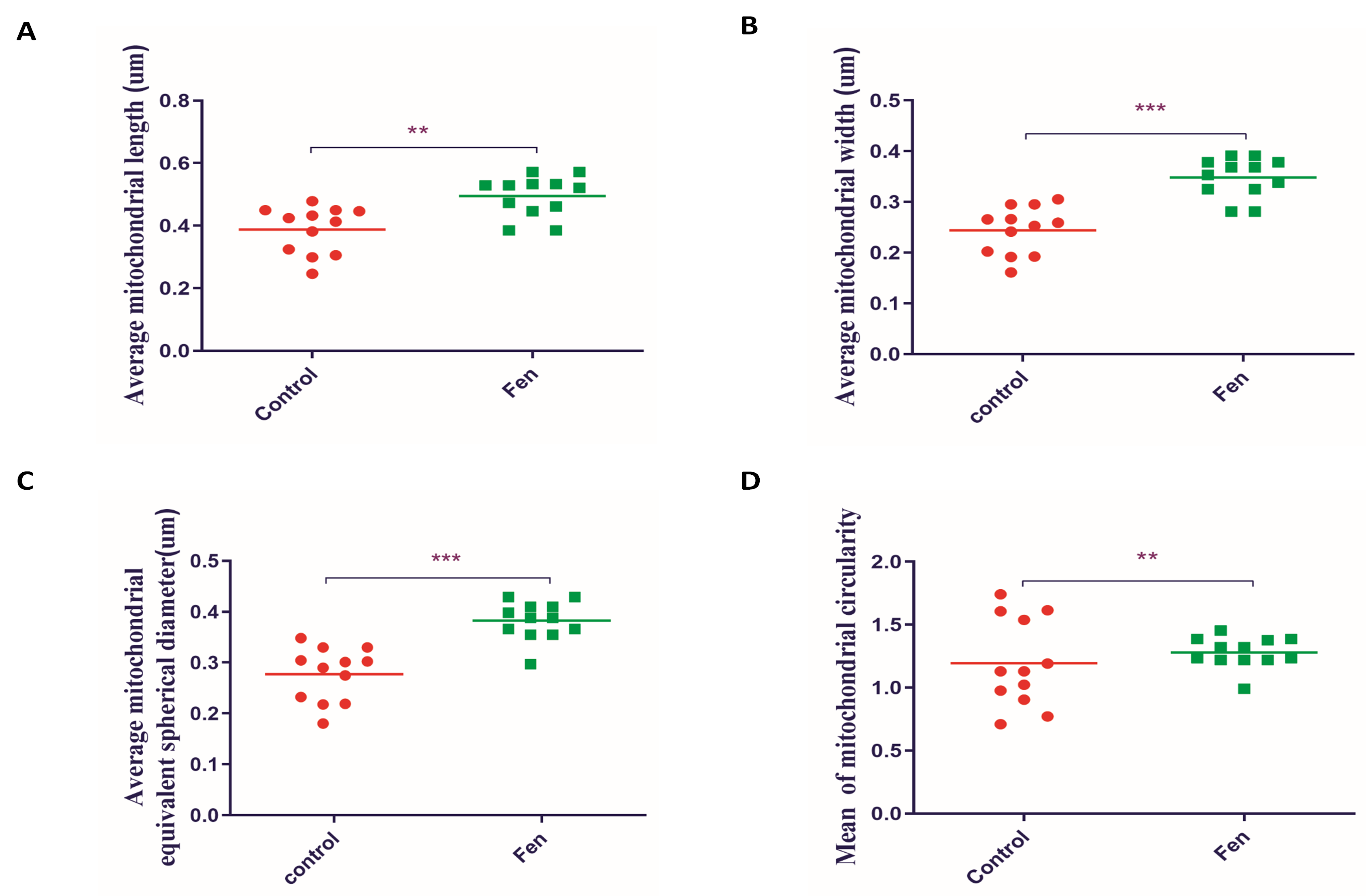

Supplement: Supplementary file 5 — Supplementary Figure 4 [file 41420_2020_313_MOESM5_ESM.tif]

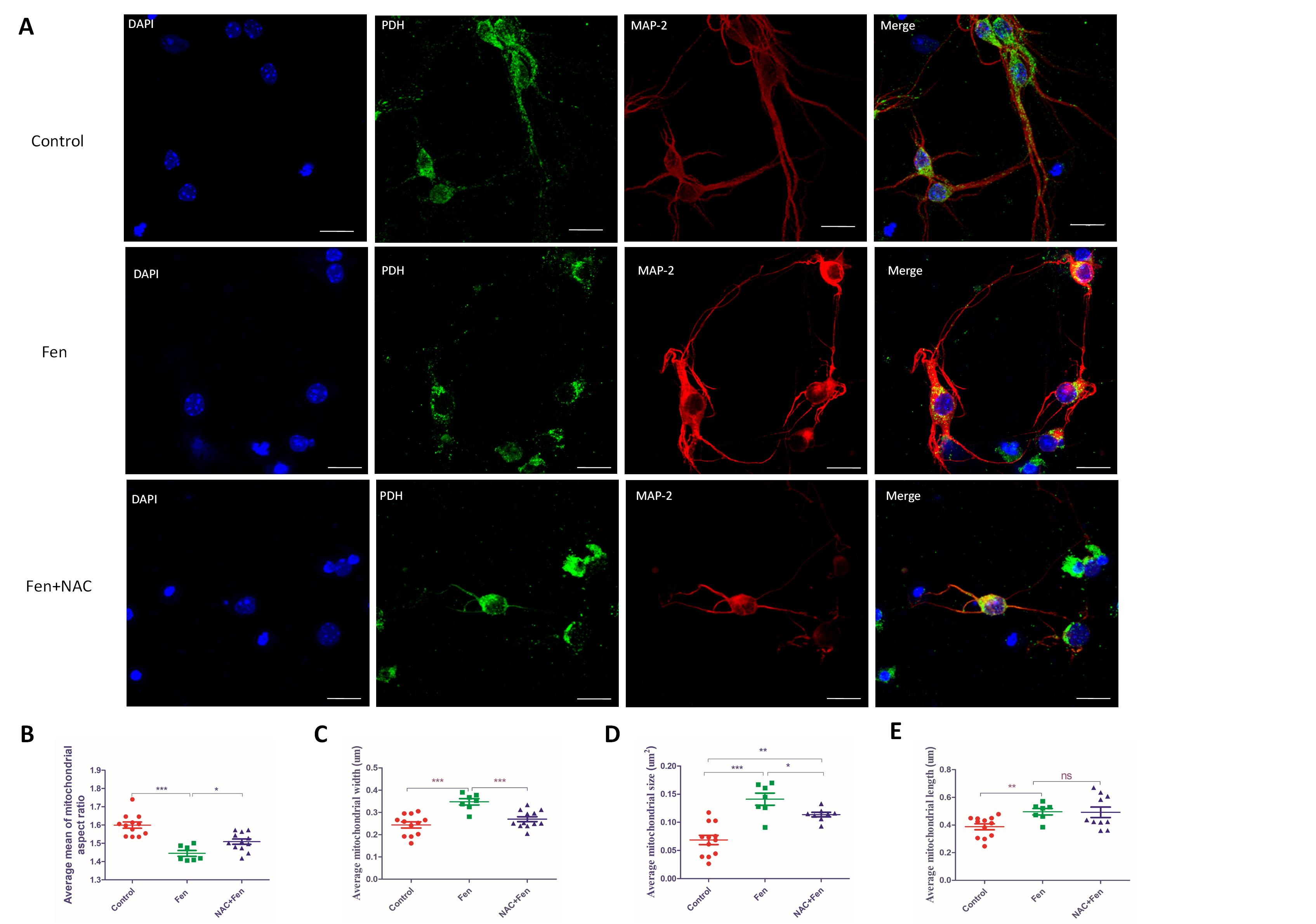

Supplement: Supplementary file 6 — Supplementary Figure 5 [file 41420_2020_313_MOESM6_ESM.tif]

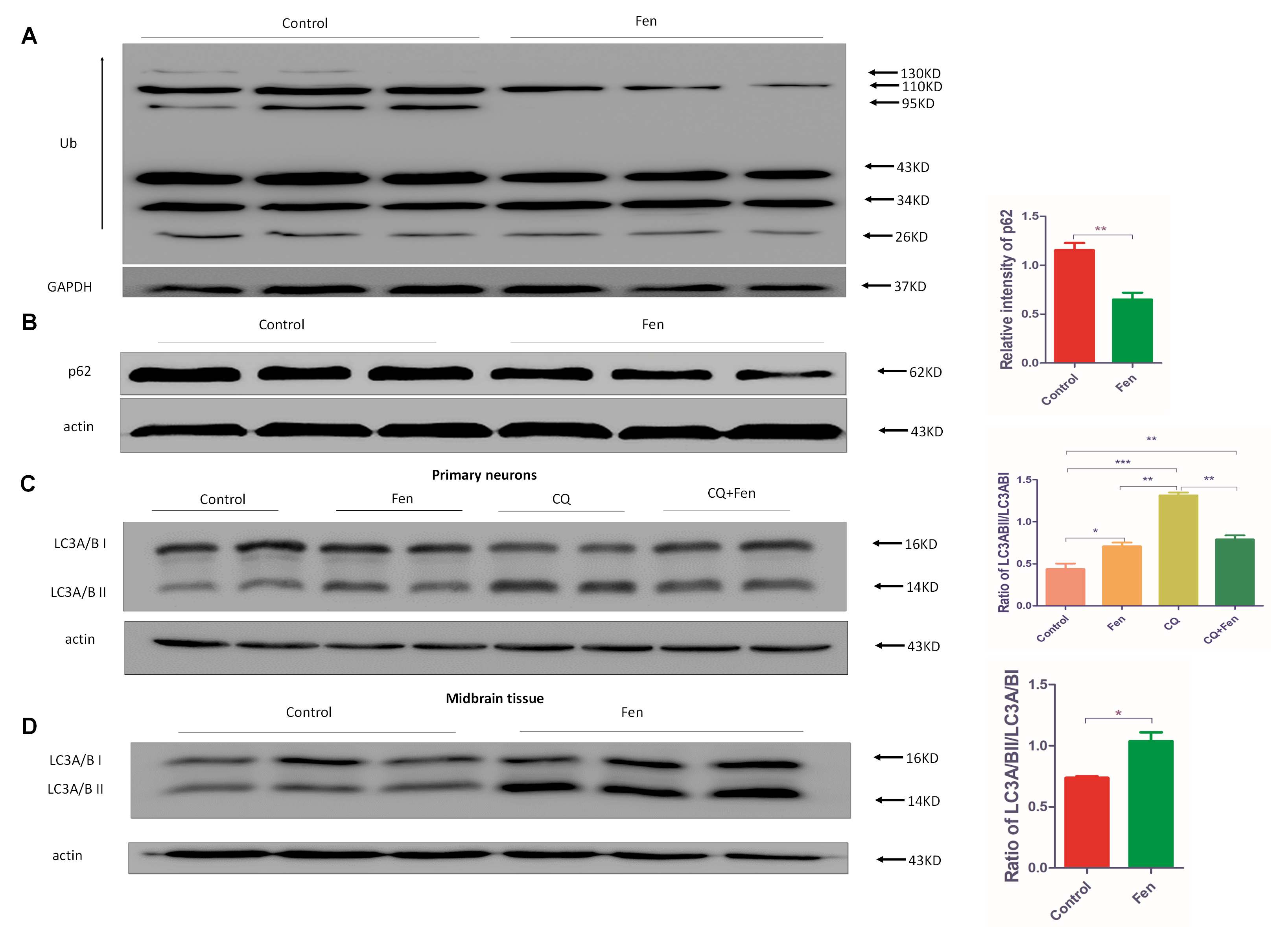

Supplement: Supplementary file 7 — Supplementary Figure 6 [file 41420_2020_313_MOESM7_ESM.tif]

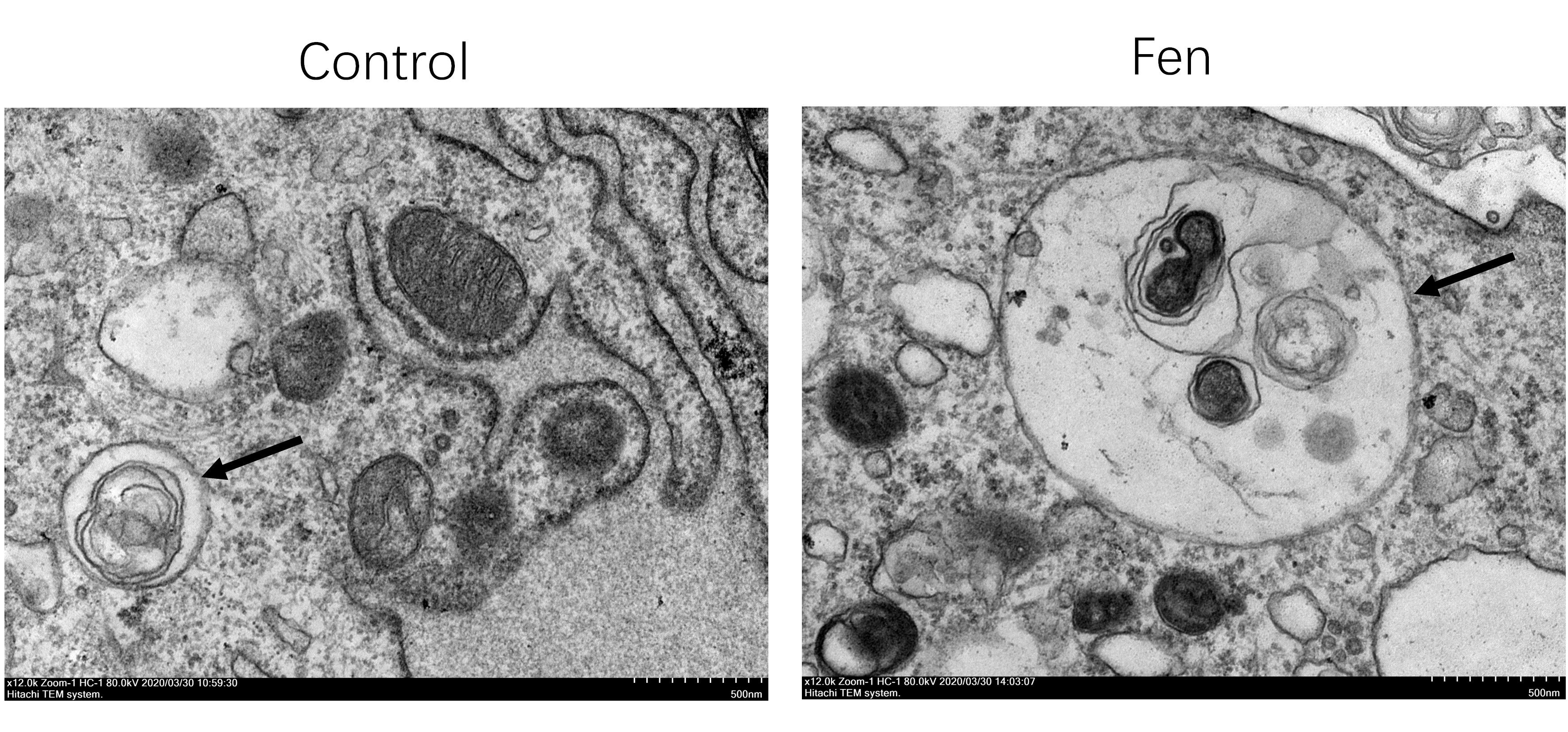

Supplement: Supplementary file 8 — Supplementary Figure 7 [file 41420_2020_313_MOESM8_ESM.tif]
